# Small incision lenticule extraction (SMILE) combined with allogeneic intrastromal lenticule inlay for hyperopia with astigmatism

**DOI:** 10.1371/journal.pone.0257667

**Published:** 2021-09-23

**Authors:** Jing Zhang, Yuehua Zhou

**Affiliations:** 1 Eye School of Chengdu University of TCM, In eye Hospital of Chengdu University of TCM, Chengdu, China; 2 Beijing Ming Vision and Ophthalmology, Dongcheng District, Beijing, China; 3 Beijing Tongren Eye Center, Beijing Ophthalmology & Visual Sciences Key Laboratory, Beijing Tongren Hospital, Capital Medical University, Beijing, China; Singapore Eye Research Institute, SINGAPORE

## Abstract

**Purpose:**

To quantitatively evaluate outcomes after small incision lenticule extraction (SMILE) combined with allogeneic intrastromal lenticule inlay for hyperopia with astigmatism.

**Methods:**

It’s a retrospective cohort study. Twenty-four eyes of 15 patients with more than 0.75 diopters (D) of astigmatism in hyperopic eyes were enrolled in this study. The hyperopic eye with astigmatism was first treated with SMILE to correct astigmatism; then a lenticule was extracted from a donor myopic eye and subsequently implanted into the hyperopic eye with astigmatism. Patients were examined preoperatively and 1 day, 1 week, 1,3 months and 1 year after surgery. The main outcome measures were the uncorrected and corrected distance visual acuity (UDVA and CDVA), uncorrected near visual acuity (UNVA), spherical equivalent (SE), corneal topography, anterior segment optical coherence topography (OCT) and ocular response analyzer (ORA) parameters: corneal hysteresis (CH) and corneal resistance factor (CRF). Repeated–measures analyses of variance (ANOVA) and post hoc tests were used to analyze data of different follow-up visits.

**Results:**

The mean preoperative cylinder was 1.95±1.04(D). The UDVA (from 0.37±0.23 to 0.09±0.09), UNVA (from 0.49±0.21 to 0.08±0.06), SE (from +7.42±3.12 to -0.75±0.79) and astigmatism (+1.95±1.04 to -0.65±0.63) postoperatively were obviously better than those before surgery. Five eyes (26.3%) gained one line of CDVA, and 3 eyes (15.8%) gained two lines of CDVA one year after surgery compared with preoperative levels. The average corneal curvature was changed from (43.19±4.37) D to (49.19±3.87) D one year after surgery. The anterior segment OCT images of corneas with lenticule inlays at each follow-up visit showed that the implanted lenticule was shaped like a crescent in the corneal stroma. The CH and CRF didn’t change significantly after surgery (p = 0.189 and p = 0.107respectively).

**Conclusions:**

SMILE combined with intrastromal lenticule inlay can be used to correct high hyperopia with astigmatism with good safety, efficacy and reproducibility.

## Introduction

There are a variety of surgical treatments available for hyperopia. Photorefractive keratectomy (PRK) and Laser Assisted In Situ Keratomileusis (LASIK) are well-known techniques for correcting hyperopia [1]. Hyperopic correction presents a different challenge to myopic correction, as regression of refractive power is a common issue post-LASIK and PRK [2]. Intrastromal lenticule implantation, which has been obtained from a myopic correction in another eye, is a newer option for hyperopia.

The implantation of a lenticule in human was first reported by Pradhan et al [3], who implanted an allogeneic lenticule obtained by a myopic SMILE procedure for correction of high hyperopia. In our previous research, allogeneic corneal small incision intrastromal lenticule inlay was used to correct hyperopic eye with good safety, effectiveness and predictability [4–6]. Other studies reported that the lenticule implantation was used for the hyperopic correction as implanting a crescentic convex-shaped lenticule obtained from a myopic SMILE procedure resulted in steeper central cornea [7–9]. Liu et al compared the postoperative higher-order-aberrations (HOAs) profiles after hyperopic SMILE, hyperopic LASIK, and lenticule implantation for correction of hyperopia, and found that lenticule implantation appeared to have less induction of HOAs in low hyperopia treatment [10]. However, to our knowledge, corneal small incision intrastromal lenticule inlay has not been explored in hyperopic eyes with astigmatism. The present study evaluated the outcomes of astigmatic hyperopic patients treated with SMILE combined with intrastromal lenticule inlay using the Visumax femtosecond system (Carl Zeiss Meditec AG, Jena, Germany).

## Materials and methods

This study was approved by the ethics committee board of the Beijing Tongren Hospital (TRECKY2014-026) and conforms with the principles and applicable guidelines for the protection of human subjects in biomedical research. Authors didn’t have access to information that could identify individual participants during or after data collection.

Twenty-four eyes of 15 Patients with more than 0.75 diopters (D) of astigmatism in hyperopic eyes were performed SMILE combined with intrastromal lenticule inlay from October 2015 to January 2019 at Beijing Tongren Hospital of Capital Medical University (Eye center, Beijing, China) and Beijing Ming Vision and Ophthalmology (Dongcheng District, Beijing, China). Informed consent was obtained from all patients in accordance with the tenets of the Declaration of Helsinki. There were 20 eyes of 12 patients followed for 1 year after surgery (3 patients were in long distance and had a teleconsultation). The mean age of the patients was 26.40±5.82 years (range from 18 to 41 years). The mean spherical equivalent (SE) was (+7.42±3.12) D (range from +4.125D to +10.375D) and the mean astigmatism was (+1.95±1.04) D (range from+0.75D to +4.25D).

### Examinations

The preoperative examinations included uncorrected distance visual acuity (UDVA), corrected distance visual acuity (CDVA) and uncorrected near visual acuity (UNVA), manifest and cycloplegic refractions, slitlamp biomicroscopy, intraocular pressure (noncontact tonometer, Canon Corp, Japan), corneal topography (TMS-4, Tomey Corp, Nagoya, Japan), corneal central thickness (CCT) (LENSTAR LS900, Switzerland) and Fourier-domain optical coherence tomography (OCT) (RTVue OCT-100, Optovue, Inc, Fremont, CA, USA). All visual acuity data were represented in the Snellen decimal system. Corneal compensated intraocular pressure (IOPcc), Goldmann correlated intraocular pressure (IOPg), corneal hysteresis (CH) and corneal resistance factor (CRF) were measured by ocular response analyzer (ORA) (Reichert, Corp, NY, USA).

The corneal topography was measured to observe changes in corneal curvature and corneal regularity by average curvature (Avek), corneal astigmatism (Cyl), surface regularity (SRI) and surface asymmetry (SAI). The anterior segment OCT was used to show the shape and position of implanted lenticule. The parameters of ORA were CH and CRF, which can be used to observe the changes of corneal biomechanics.

Measurements of UDVA, UNVA, CDVA, manifest refraction, and OCT were obtained at 1 day, 1 week, 1, 3 months and 1 year after surgery. The corneal topography, CCT and ORA were measured at 1, 3 months and 1 year after surgery.

### Surgical technique

All surgical procedures were performed by one experienced SMILE surgeon (Y.H.Z.) using topical anesthesia as described. Prior to the donors’ SMILE surgery, the baseline work-up of the donors had been completed in Beijing Tongren Hospital to test for HIV, Hepatitis B and C, and Treponema pallidum particle agglutination assay.

To begin with, hyperopic astigmatism eye underwent SMILE procedure with -0.50D myopia and astigmatism with a 2-mm and 90 ◦ incision, and the thickness of the cap was 110um. (Optical zone 6.5mm, Cap diameter 7.0mm, Hinge angle 90◦, energy offset 140nJ, and spot separations lenticule 4.5um, lenticule side 2.0um). In addition, the patient with a myopic refractive error corresponding to the absolute value of residual hyperopia was scheduled for a routine SMILE procedure (Optical zone 6.5mm, Cap diameter 7.5mm, Hinge angle 90◦) on the same day and immediately after the recipient patient with hyperopic astigmatism. The lenticule was set aside in equilibrium liquid and stained with 0.1% riboflavin solution (Medio-Haus Medizinprodukte GmbHBrunswikerStraBe 50, Kiel, Germany) for 1 minute for isocentric localization. Then, the donor lenticule was immediately inserted into the pocket of the hyperopic patient through the small incision using a Kelman forceps. The donor lenticule was flattened at the corneal center of the recipient eye. The diameter of lamellar pocket (7.0mm) was 0.5mm more than that of the donor lenticule (6.5mm). Postoperatively, loteprednol 0.1% was used four times a day for 1 week which was subsequently reduced to one time every week for 1 month. Levofloxacin 0.3% and artificial tears were used four times per day for 2 weeks.

### Statistical analysis

Statistical analysis was performed using SPSS software (Version 19.0, SPSS Inc., Chicago, IL, USA). The Shapiro-Wilk test was used for normal distribution analysis. Repeated–measures analyses of variance (ANOVA) and post hoc tests were used to analyze data of different follow-up visits. In the groups not showing normal distribution, Wilcoxon test was used. A P value < 0.05 was considered as statistically significant.

## Results

There were no significant intraoperative or postoperative complications such as inflammation or infection, haze, decentered lenticule and epithelial ingrowths. All corneas of 12 patients remained transparent without decentered lenticule observed under the slip lamp.

### Visual acuity and refraction

**Fig 1:**
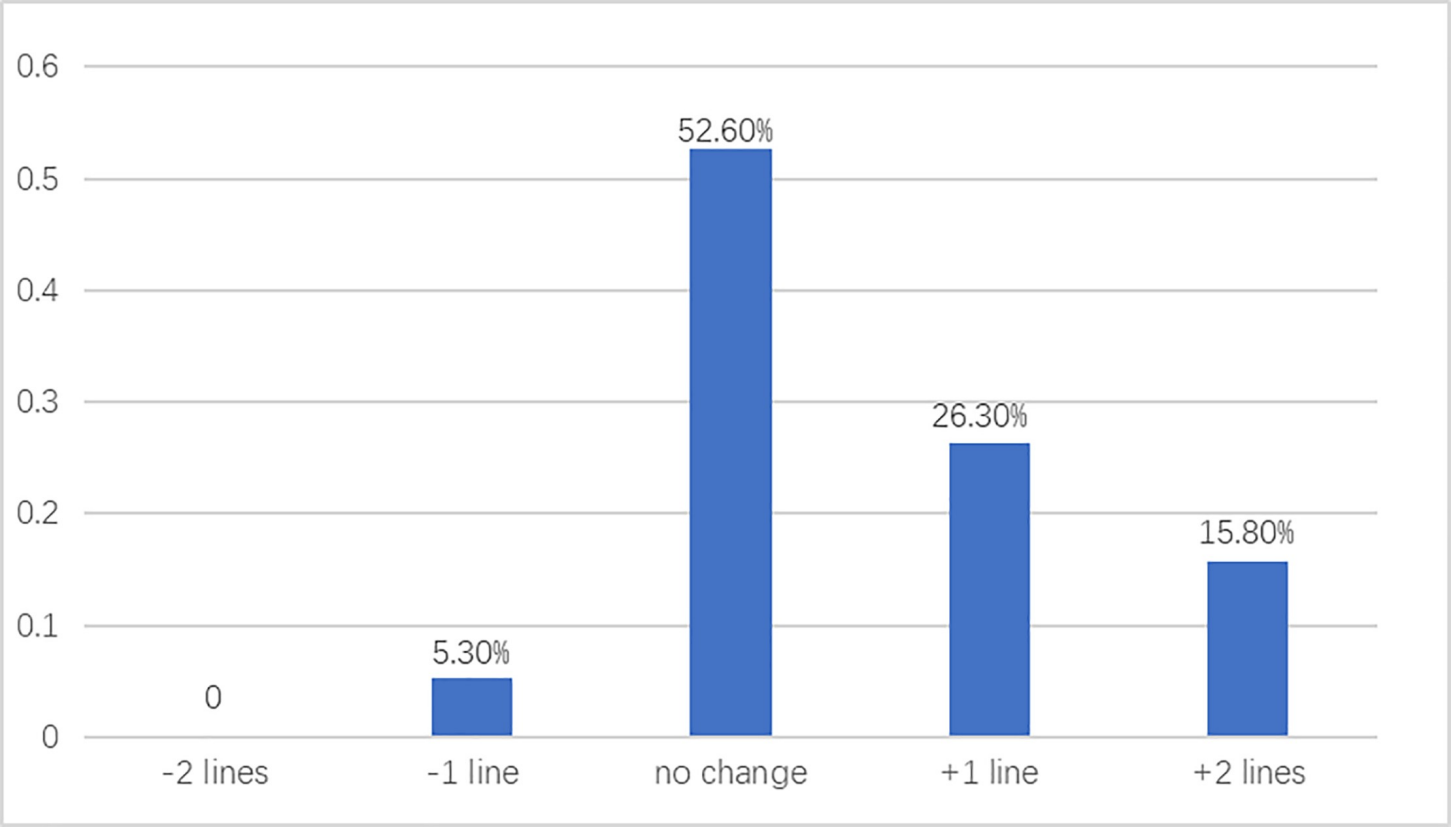
The change of CDVA before and one year after surgery.

The changes of visual acuity and refraction results are listed in Table 1. The UDVA, UNVA, SE and astigmatism at each follow-up visit postoperatively were better than those before surgery (p all <0.01). CDVA remained unchanged (pre-OP: 0.09 ± 0.07; post-OP: 0.08 ± 0.07; p = 0.421). The mean SE was changed from (+7.42±3.12) D preoperatively to (-1.91±0.56) D and astigmatism was changed from (+1.95±1.04) D preoperatively to (-2.53±2.28) D one day after surgery (p all <0.01). The results showed mild overcorrection states in the earlier postoperative stages (about 1 week) while the overcorrection subsided at 1 month and stabilized thereafter (Table 1). There was only one eye lost one line of CDVA (5.30%) and no eye lost more than one line of CDVA. Five eyes (26.3%) gained one line of CDVA, and 3 eyes (15.8%) gained two lines of CDVA one year after surgery compared with preoperative levels (Fig 1).

**Table 1:** Visual acuity and refraction before and after surgery.

	UDVA	UNVA	CDVA	SE(D)	Astigmatism(D)
Before	0.37±0.23*	0.49±0.21*	0.09±0.07	+7.42±3.12*	1.95±1.04*
1 day	0.19±0.17	0.18±0.12	0.08±0.09	-1.91±0.56	-2.53±2.28
1 week	0.17±0.16	0.09±0.07	0.07±0.11	-1.12±1.71	-1.83±1.67
1 month	0.14±0.15	0.07±0.10	0.09±0.10	-0.85±0.86	-1.08±1.66
3 months	0.12±0.12	0.06±0.08	0.07±0.06	-0.76±0.69	-0.97±0.58
1 year	0.09±0.09	0.08±0.06	0.08±0.07	-0.75±0.79	-0.65±0.63
F	9.387	41.379	1.483	52.376	19.264
p	p<0.01	p<0.01	0.421	p<0.01	p<0.01

*D* = diopter; *UDVA* = uncorrected distance visual acuity; *UNVA* = uncorrected near visual acuity; *CDVA* = corrected distance visual acuity; SE = spherical equivalent.

*p<0.01

### Corneal topography and CCT

The average corneal curvature was changed from (43.19±4.37) D to (49.79±3.96) D one-month after surgery (p<0.01), then corneal curvature stabilized thereafter. SRI and SAI were increased one month after surgery (p<0.01), which changed from (0.28±0.21) and (0.65±0.37) to (0.76±0.65) and (1.10±0.72) one month after surgery. The central corneal thicknesses were increased one month after surgery (p<0.01). There were no significant differences in central thickness and corneal curvature compared with 1 month and 1 year postoperatively (pall> 0.05) (Table 2).

**Table 2:** Corneal topography and CCT before and after surgery.

	Avek	Cyl	SRI	SAI	CCT
Before	43.19±4.37*	2.72±1.38*	0.28±0.21*	0.65±0.37*	529.25±41.25*
1 month	49.79±3.96	1.17±0.75	0.76±0.65	1.10±0.72	558.04±38.59
3 months	49.46±4.21	1.28±0.62	0.83±0.57	1.17±0.65	555.51±42.37
1 year	49.19±3.87	1.34±0.59	0.76±0.49	1.18±0.68	556.59±36.91
F	24.75	12.67	21.53	22.11	16.19
P	p<0.01	p<0.01	p<0.01	p<0.01	p<0.01

Avek = average curvature; Cyl = corneal astigmatism; SRI = surface regularity index; SAI = surface asymmetry index; CCT = central corneal thickness.

*p<0.01

### Anterior segment OCT

**Fig 2:**
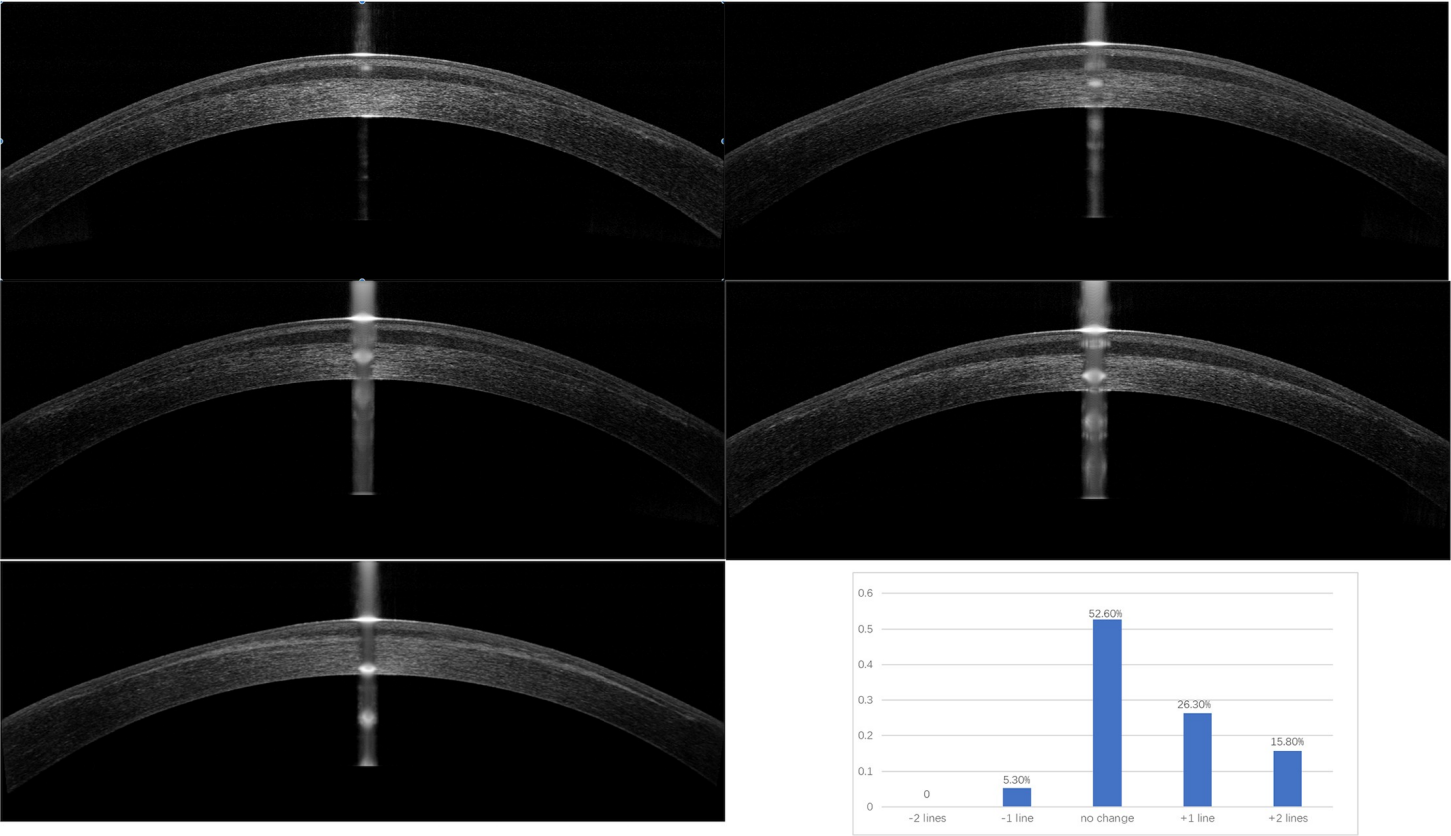
Anterior segment OCT images of corneas with lenticule implantation at each follow-up visit. Pictures a-e respectively represents OCT images 1 day, 1 week, 1 month, 3 months and 1 year after surgery. The implanted lenticule was in the center and shaped like a crescent in the corneal stroma, with no offset and wrinkles at each visit. The lenticule demarcation line blurred at 1 year after surgery.

The anterior segment OCT images of corneas with lenticule inlays at each follow-up visit showed that the implanted lenticule was in the center and shaped like a crescent in the corneal stroma, with no offset and wrinkles. As time passed, the lenticule demarcation line blurred gradually (Fig 2). The thickness of lenticule was measured from 1 month after surgery and the thickness of lenticule was stable (1-month pos-OP: 94.71±24.48 um, 1-year pos-OP: 94.05±24.54 um, p = 0.892).

### Corneal biomechanics

The IOPg and IOPcc were obviously decreased one-month after surgery (p<0.01 and p = 0.03 respectively) compared with those preoperatively. While there were no significant differences in IOPg and IOPcc compared with 1 month and 1 year postoperatively (p all> 0.05). The CH and CRF didn’t change significantly after surgery (p = 0.189 and p = 0.107 respectively) (Table 3).

**Table 3:** Corneal Biomechanics before and after surgery.

	IOPg	IOPcc	CH	CRF
Before	13.89±3.37*	12.98±3.95*	11.28±2.09	11.74±2.13
1 month	11.63±2.71	12.05±4.39	10.74±1.85	11.26±1.85
3 months	11.89±2.86	11.73±3.61	11.93±2.17	11.92±2.01
1 year	12.19±2.93	12.57±2.96	11.65±2.26	12.35±1.69
F	4.129	3.987	1.548	1.872
P	P<0.01	0.03	0.189	0.107

IOPcc = Corneal compensated intraocular pressure; IOPg = goldmann correlated intraocular pressure; CH = corneal hysteresis; CRF = corneal resistance factor.

*p<0.05

## Discussion

In the present, the surgical correction of hyperopia, especially hyperopia with astigmatism has lagged behind the surgery of myopia in terms of predictability and long-term stability, partly because it is much more difficult to surgically steepen the cornea to correct hyperopia than it is to flatten the cornea to correct myopia [11–13]. Convincing data about the accuracy and stability for a long period of the refractive correction has been obtained using SMILE for myopia [14–17]. Several studies have showed that corneal small incision intrastromal lenticule inlay can be used to correct hyperopic eye with good safety, effectiveness and predictability [3–10]. Meanwhile new treatments for hyperopia are emerging [18]. As a continuity of allogeneic lenticule implantation for hyperopic research, we performed this study to evaluate outcomes of SMILE combined with allogeneic corneal intrastromal lenticule inlay for hyperopia with astigmatism, based on our previous study.

SMILE combined with allogeneic corneal intrastromal lenticule inlay for hyperopic astigmatism was safe. There were no side effects after the implantation of donor tissue. At the one-year follow-up visit, all the corneas of 12 patients remained transparent, without intraoperative or postoperative complications such as inflammation or infection, haze, decentered lenticule and epithelial ingrowths. Furthermore, no eye lost more than 1 line of CDVA.

A donor lenticule created during SMILE procedure can be implanted into the recipient eye through a 2mm incision with a stromal delamination pocket to correct hyperopia. As the donor lenticule without blood vessels and lymphatic tissue is in a sterile corneal capsular bag, and not in contact with the aqueous humor and blood, the probability of corneal rejection and infection is extremely low. Even in the event of corneal rejection and infection, we can remove the corneal donor lenticule and replace it [6]. In our study, the corneas remained clear over the 1-year postoperative period, but longer follow-up periods are needed because tissue rejection can occur even several years postoperatively, as has been observed after corneal transplantation [19].

Improvements of UDVA and UNVA are important indicators to measure the effectiveness of this surgery. In this study, we found that the UNVA (from 0.49 to 0.08) and SE (from +7.42D to -0.75D) significantly improved one year after surgery compared with those preoperatively, which proved that SMILE with the allogeneic lenticule inlay had good effectiveness. Our results showed that the improvement of UNVA was more significant than that of UDVA (Table 1), which has an important significance for near distance workers and seniors. Five eyes (26.3%) gained one line of CDVA, and 3 eyes (15.8%) gained two lines of CDVA one year after surgery compared with preoperative levels. There was only one eye lost one line of CDVA (5.30%) and no eye lost more than one line of CDVA. The postoperative wound healing might contribute to one line lost of CDVA. Ganesh et al [7] also reported that 4 of 9 eyes gained better CDVA after allogeneic lenticule implantation. In this study, we believe that improved CDVA might be related to the improvement of visual performance and further study is needed regarding this aspect.

The most critical step in SMILE with allogeneic lenticule implantation to correct hyperopia with astigmatism was to guarantee center alignment during operation. In the current research, the corneal vertex of the coaxially fixating eye was aligned with the vertex of the curved contact glass. Furthermore, the diameter of lamellar pocket was 0.5mm more than that of the donor lenticule, which is consistent with Reinstein DZ’s research [20] and the donor lenticule was stained with riboflavin solution for isocentric localization.

In our study, the average corneal curvature was changed from (43.19±4.37) D to (49.79±3.96) D 1-month after surgery and the corneal curvature was stable after 1 month postoperatively. While Ganesh et al. [7] reported a fairly good refractive predictability using this technique to treat moderate hyperopia, although results showed that this technique were not highly accurate for correcting very high hyperopia. Sun et al. [8] also demonstrated that refractive predictability was unsatisfactory after autologous lenticule transplantation, which may have been related to the shape of lenticule following astigmatism correction. We speculate that epithelial remodeling, anterior and posterior corneal surface changes, and postoperative wound healing contributed to the changes of results. Moreover, refractive predictability after lenticule re-implantation requires further research.

The results showed that most eyes were in a mild overcorrection state in the earlier postoperative stage (about 1 week) but the overcorrection subsided at 1 month and stabilized thereafter. This may have occurred because the hyperopic astigmatic eye may have over-accommodated for a long period [21, 22]. Although this surgery corrected the refractive error, the over-accommodation continued during the early stage after surgery and patients need more time to adapt to the new refractive state; and as time passed, the accommodation relaxed and overcorrection disappeared gradually [8]. Ganesh et al. [7] and Williams GP et al. [23] reported that epithelial remodeling after lenticule implantation could be one of the reasons for immediate overcorrection during the first month. Another explanation was that over-myopia had to do with the edema of the lenticule and once swelling of the tissue disappeared, there was reduction of myopia and the eye returned to the emmetropia. In the current study, there were no significant differences in SE, astigmatism, central corneal thickness and corneal curvature compared with 1 month, 3 months and 1 year postoperatively, which proved that SMILE combined with allogeneic lenticule inlay for hyperopia with astigmatism had good stability. However, several studies of femtosecond laser-assisted LASIK showed regression after surgery [24, 25] and therefore further and longer-duration studies are needed for this aspect.

In addition, the incisions in the SMILE program were only 2-mm above the cornea, so the corneal biomechanics was retained after surgery. There were no significant differences in CH and CRF l year postoperatively compared with preoperatively. SMILE combined with allogeneic lenticule inlay for hyperopic astigmatism correction possibly protects nerve fibers within the corneal stroma, and therefore could reduce the incidence of dry eye and corneal flap complications.

In conclusion, SMILE combined with allogeneic corneal intrastromal lenticule inlay can be used to correct hyperopic astigmatic eye with good safety, efficacy and reproducibility. In another way, we could simply insert a lenticule with a higher amount of hyperopic correction, let the correction stabilize and then perform a myopic astigmatic correction in the form of PRK for the residual astigmatic myopic refractive error. Further research with studies of longer-durations and larger-sample sizes are yet to be done.

## Supporting information

S1 Fig (TIF)

S1 Data (XLS)
