# Supplementary figures and images for: Small incision lenticule extraction (SMILE) combined with allogeneic intrastromal lenticule inlay for hyperopia with astigmatism
Source: PLoS One. 2021 Sep 23;16(9):e0257667. doi: 10.1371/journal.pone.0257667 (PMC8460088; doi:10.1371/journal.pone.0257667)

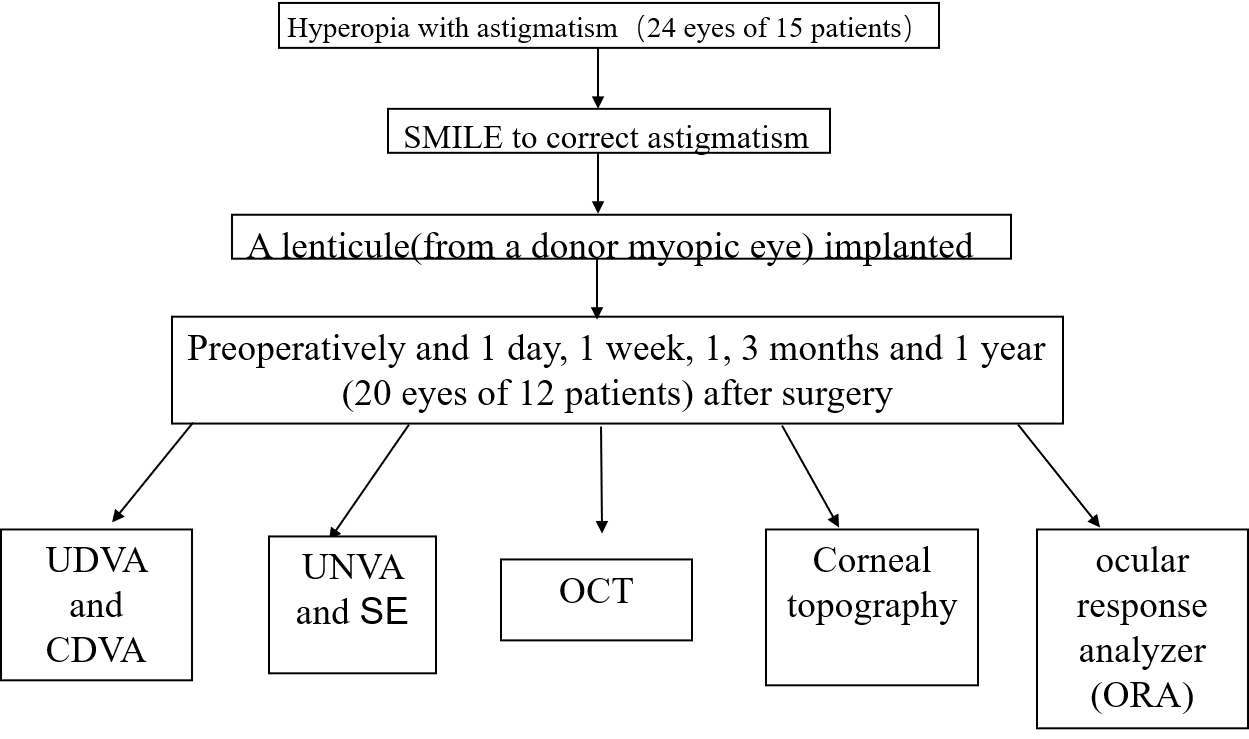

Supplement: S1 Fig — (TIF) [file pone.0257667.s001.tif]
